# Clinical efficacy of Masquelet technique in the treatment of tibial and fibular bone defects caused by severe gunshot wounds

**DOI:** 10.3389/fmed.2026.1889353

**Published:** 2026-08-28

**Authors:** Qiming Sun, Yingjie Cong, Wei Sun, Xinwei Liu, Baolei Guo, Chunli Zhao, Baoxian Hou, Ye Liu, Dapeng Zhou

**Affiliations:** 1 Department of Burns and Plastic Surgery, General Hospital of Northern Theater Command, Shenyang, Liaoning, China; 2 Department of Hand and Foot Microsurgery, Shenyang Orthopedic Hospital, Shenyang, Liaoning, China; 3 Department of Teaching and Research, Shenyang Orthopedic Hospital, Shenyang, Liaoning, China

**Keywords:** bone defect, gunshot wound, Masquelet technique, open tibiofibular fracture, segmental bone

## Abstract

**Objective:**

To explore the clinical efficacy of the Masquelet technique in the treatment of tibial and fibular bone defects resulting from severe gunshot wounds.

**Methods:**

A retrospective analysis was conducted on six male patients (mean age, 23 ± 5 years; range, 18 to 33 years) with tibial and fibular bone defects resulting from gunshot wounds, treated with the Masquelet technique between January 2022 and January 2024. In Stage 1, blood transfusions and fluid resuscitation were administered to manage shock. Wound debridement was performed, followed by applying an external fixator. The average length of the bone defects was 8.7 ± 2.2 cm (range, 5.4 to 12.3 cm). The bone defect areas were filled with vancomycin-loaded bone cement, and the wounds were closed using vacuum sealing drainage (VSD). In Stage 2, the bone cement device was surgically removed, and the external fixator was replaced with an intramedullary nail. Autologous iliac bone grafts in strip form were implanted in the induced membrane to manage the bone defects. Postoperatively, the wound healing and inflammatory indicators were observed to determine the infection control status. Regular X-ray examinations on the affected extremities were conducted to evaluate the bone healing status in the defect areas.

**Results:**

The mean follow-up duration was 13.7 ± 0.9 months (range, 12 to 15 months). The interval between Stage 1 and Stage 2 ranged from 16 to 32 weeks (mean 22.7 ± 5.1 weeks). The number of debridement in Stage 1 varied from 2 to 3 times (mean 2.5 ± 0.5 times). All six patients underwent free flap transplantation for wound coverage, and the flaps healed well in all patients. Bone union was achieved in all six patients, and the healing time was 10 to 15 months (mean 12.5 ± 1.5 months). Callus formation was uniform in the bone defect areas, indicating successful bone union, and the fracture lines became blurred. No major complications, such as deep infection, osteomyelitis, hardware failure, or re-fracture, were observed during the follow-up period.

**Conclusion:**

The Masquelet technique demonstrates favorable therapeutic effects on tibial and fibular bone defects resulting from severe gunshot wounds, and it represents a safe and reliable method for addressing such complex bone defects. However, given the small sample size and retrospective design of this study, further large-scale prospective studies are warranted to confirm these findings.

## Introduction

Gunshot wounds, as severe open injuries, occur relatively infrequently in China, but are more commonly encountered in international conflicts, humanitarian aid missions, and military operations. Gunshot wounds often result in complex fractures and severe soft tissue damage, with the management of bone defects posing a particular challenge (1, 2). Traditional approaches for the treatment of bone defects include vascularized bone grafting, the Ilizarov technique, and the Masquelet technique (3, 4). However, to date, there is no unified standard of care that can effectively address the challenges posed by gunshot wounds (5). The Masquelet induced membrane technique (MIMT) is a relatively new, two-stage surgical procedure for the reconstruction of segmental bone defects, which was innovatively proposed by Dr. Masquelet in the mid-1980s (6). Numerous studies have demonstrated its great potential in treating large bone defects and it has become one of the preferred methods for repairing severe bone defects in the extremities (7). Compared to the traditional distraction osteogenesis(DO) technique, MIMT has shown better outcomes in treating critical-sized bone defects and has also achieved success in cases involving large bone defects exceeding 15 cm (8). This method has been used to bridge diaphyseal defects of up to 25 cm in length (5). Although MIMT has been used worldwide, to date, only a few studies have reported on the use of the Masquelet technique for the reconstruction of combat-related bone defects (1, 7–10). Research on its application in tibial and fibular bone defects resulting from gunshot wounds remains scarce. The current study aimed to retrospectively analyze and explore the clinical efficacy of the Masquelet technique for the treatment of tibial and fibular bone defects caused by severe gunshot wounds, in order to provide an economical, reliable, and highly effective treatment method in this field.

## Materials and methods

### General data

This study was a retrospective analysis of six male patients with tibial and fibular bone defects caused by severe gunshot wounds who underwent treatment using the induced membrane technique between January 2022 and January 2024. The patients’ ages ranged from 18 to 33 years, with an average age of 23 ± 5 years. According to the Gustilo-Anderson classification of open fractures, all patients had Gustilo Type III open injuries (5 patients with Gustilo IIIC and one patient with Gustilo IIIB). All six patients presented with local skin defects, and comminuted tibiofibular fractures with bone exposure. Five patients had rupture of the anterior tibial artery, with the posterior tibial artery remaining intact, while the remaining one patient had both the anterior tibial artery and the posterior tibial artery intact. After intraoperative debridement, the bone defects in six patients ranged in length from 5.4 to 12.3 cm, with an average length of 8.7 ± 2.2 cm. Postoperatively, all six patients underwent free flap transplantation to cover the wounds. A summary of patient demographics and clinical characteristics is presented in Table 1.

**Table 1:** Patient demographics and clinical characteristics.

Patient	Age (years)	Gustilo type	Bone defect length (cm)	Artery injury	Flap type	Follow-up (months)
1	21	IIIC	7.8	Anterior tibial artery rupture	Anterolateral thigh flap	14
2	18	IIIB	5.4	Both intact	Anterolateral thigh flap	14
3	33	IIIC	9.3	Anterior tibial artery rupture	Anterolateral thigh flap	13
4	22	IIIC	12.3	Anterior tibial artery rupture	Anterolateral thigh flap	15
5	25	IIIC	10	Anterior tibial artery rupture	Anterolateral thigh flap	14
6	19	IIIC	7.5	Anterior tibial artery rupture	Anterolateral thigh flap	12

### Inclusion and exclusion criteria

Inclusion criteria included patients (1) open tibiofibular fractures with bone defects caused by gunshot wounds; (2) patients without life-threatening conditions after emergency treatment and eligible for the second stage surgery; (3) patients aged between 18 and 60 years; (4) patients who voluntarily signed the informed consent form for surgery and underwent surgery.

Exclusion criteria included patients (1) patients who died despite emergency treatment or underwent amputation due to severe gunshot wounds; (2) patients with injured limbs accompanied by nerve damage, resulting in postoperative loss of function; (3) patients with short follow-up periods or those lost to follow-up; (4) patients who refused to sign the informed consent form for surgery.

### Preoperative preparation

The anti-shock treatment after admission was aggressively performed in all patients. After the vital signs of the patients stabilized (3 h after admission), computed tomography angiography (CTA) of the arteries of both lower extremities was performed immediately. Routine preoperative blood tests such as routine blood tests and biochemical examination, electrocardiographic, X-ray, and computed tomography (CT) examinations were conducted. The damage to the blood vessels of both lower limbs was determined based on the preoperative examination. If severe arterial injury was present and hemostasis could not be achieved, emergency amputation surgery was required. After the shock was corrected, the scope of bone removal during surgery was determined based on preoperative CT findings.

### First stage (debridement, fixation, and skin flap transplantation)

All patients underwent debridement of open wounds under general anesthesia. The operative field was thoroughly flushed with a large amount of saline, and the skin edges were trimmed until they were expanded and necrotic skin edges were excised by 2 mm. Necrotic and contaminated tissues, as well as devitalized and necrotic muscles, were removed. During surgery, multiple-site sampling was conducted on the target area for bacterial culture and pathological examination; postoperative sampling was not performed. Three or more deep tissue or fluid samples were collected when sampling. Dedicated surgical instruments were used for handling the obtained samples to avoid cross-contamination during sampling. Superficial wounds or epidermal samples were avoided from being collected. Necrotic tendons, blood vessels, and nerves were managed according to the guidelines for open injury treatment (11). Bone tissue was debrided until fresh blood oozed from the cortical bone (paprika sign). After debridement was completed, dilute povidone-iodine solutions and large amounts of normal saline were used to flush the operative field. Then, the operative field was re-disinfected, additional sterile drapes were placed, and the surgical personnel changed their surgical gloves and gowns. Composite external fixation frame was used to fix the fracture ends, maintaining the alignment and length of the lower limbs. Antibiotic-impregnated bone cement bead chains were prepared for wound coverage by mixing polymethyl methacrylate (PMMA) bone cement with vancomycin (adding 4 g of vancomycin per 40 g of PMMA). Bone cement spacers were inserted in the bone defects, which covered an additional 1 to 2 cm on both sides of the bone defect area. When making tibial bone defect spacers, a 50-ml syringe could be used for shaping to make the surface of the bone cement smooth. During the heat dissipation of bone cement, saline should be used to cool it down to prevent the surrounding soft tissue from being burned. The VSD device was used to cover and drain the wounds in the first stage. One week later, based on the intraoperative and postoperative bacterial culture results and pathological findings, the antibiotic-impregnated bone cement was replaced if infection was not controlled or there was persistent exudate; otherwise, the cement spacer was retained until the second stage. All six patients required the second debridement surgery and were performed until there were no infected or necrotic tissues within the surgical site, the wound was dry with no inflammatory exudation, and the granulation tissue was fresh. During surgery, soft tissue and bone tissue samples were taken for bacterial culture and pathological examination, both yielding negative results. Additionally, blood test indicators such as white blood cell count, C-reactive protein (CRP), procalcitonin (PCT), and erythrocyte sedimentation rate (ESR) were verified to be within normal ranges before proceeding to replace the external fixation with the antibiotic cement-coated intramedullary nail to fix the fracture ends. Free skin flap transplantation was performed to cover the wound. Once the wound healed satisfactorily, and all indicators from repeated blood tests, bacterial cultures, and pathological examinations were confirmed to be within normal ranges, the second-stage surgery could be performed.

In this study, due to the high degree of contamination and severity of the gunshot wounds, the infection control process required more time than usual. Although the idealized timing for the second-stage surgery is 6 to 8 weeks after the first stage, all six patients required additional debridement procedures (2 to 3 times in Stage 1) to achieve complete infection control. Consequently, the actual interval between the first and second stages was prolonged to 16 to 32 weeks (mean 22.7 ± 5.1 weeks).

### Second stage (bone grafting)

The second-stage surgery was conducted after complete infection control and satisfactory soft tissue healing had been achieved. Although the conventional recommendation is 6 to 8 weeks following the first-stage surgery, in this series, the actual interval was 16 to 32 weeks (mean 22.7 weeks) due to the need for multiple debridement procedures to achieve infection control. During this period, free or pedicled myocutaneous flap transplantation could be conducted to cover the wound. The bacterial culture and pathological results after debridement were both negative; preoperative inflammatory indicators such as white blood cell count, C-reactive protein (CRP), procalcitonin (PCT), and erythrocyte sedimentation rate (ESR) were all normal. The second-stage surgery could be performed following complete healing of the flaps or wounds, with no wound exudation or large scab formation in the local wounds, and after the patients were monitored for three days with no fever, pain in the affected limbs, and other adverse symptoms. A longitudinal incision was made on the surface of the induced membrane to completely remove the bone cement spacer and resect the sclerotic bone at the fracture ends. Subsequently, iliac bone grafting was performed (when the length of the bone defect was large, both anterior and posterior iliac bone harvesting should be performed simultaneously). If the stability of the fracture ends was insufficient, the monocortical bone plate could be used for secure fixation to promote healing of the bone defect.

### Postoperative treatment

The drainage tube was routinely inserted into the surgical incision for 48 h postoperatively, which was removed when the drainage volume was less than 200 mL/day. The duration of systemic antibiotic administration postoperatively was 6 to 8 weeks. Weight bearing on the operated limb was prohibited, and regular radiological examinations of the operated limb were conducted. The functional training for the lower extremities was adjusted based on the healing progress observed in imaging studies.

### Follow-up and outcome measures

*Main outcome measures*: The rate of fracture healing was evaluated, and the patients who were still unhealed 9 months after surgery and showed no signs of progressive healing were classified as non-union. Fracture healing included clinical union and bone union.

*Criteria for clinical union of bones*: (1) no tenderness and longitudinal percussion pain in local part of the fracture site; (2) no abnormal local activity; (3) X-ray showed continuous callus at the fracture site, and the fracture line became blurred; (4) ability to walk continuously for three minutes (not less than 30 steps) without external fixation; (5) no deformation at the fracture site for two consecutive weeks.

*Criteria for bone union of fractures*: (1) meeting the conditions for clinical union of bones; (2) X-ray showed bone trabeculae crossing the fracture line.

The trend of changes in the patients’ serological indicators of infection was monitored weekly after surgery. When infection was suspected, sensitive antibiotics should be replaced according to the drug sensitivity test of intraoperative bacterial culture. The X-ray examination was performed every 4 weeks to evaluate the bone healing and record the bone healing time. The follow-up period after bone healing was changed to once every 12 weeks.

### Statistical analysis

Data were analyzed using SPSS 26.0. Due to the retrospective case series design and small sample size (n = 6), descriptive statistics were primarily used to summarize the data. Continuous variables were expressed as mean ± standard deviation (SD) and range. Categorical variables were presented as frequencies and percentages. No comparative statistical tests were conducted due to the absence of a control group.

## Results

All the patients were followed up for 12 to 15 months, with an average of 13.7 ± 0.9 months. The interval between Stage 1 and Stage 2 ranged from 16 to 32 weeks, with an average of 22.7 ± 5.1 weeks. The number of debridement in Stage 1 varied from 2 to 3 times, with an average of 2.5 ± 0.5 times. All six patients underwent free flap transplantation for wound coverage, and the flaps healed well in all patients. Bone union was achieved in all six patients, and the healing time was 10 to 15 months, with an average of 12.5 ± 1.5 months. Callus formation was uniform in the bone defect areas, indicating successful bone union, and the fracture lines became blurred.

Regarding postoperative complications, no deep infections, osteomyelitis, hardware failure, or re-fracture were observed during the follow-up period. All flaps survived completely without necrosis or significant wound dehiscence. Superficial wound infections occurred in two patients (33.3%), which were successfully managed with local wound care and sensitive antibiotic therapy without affecting the final bone healing outcomes. No patient required amputation or additional unplanned surgical intervention for infection control.

## Discussion

**Figure 1:**
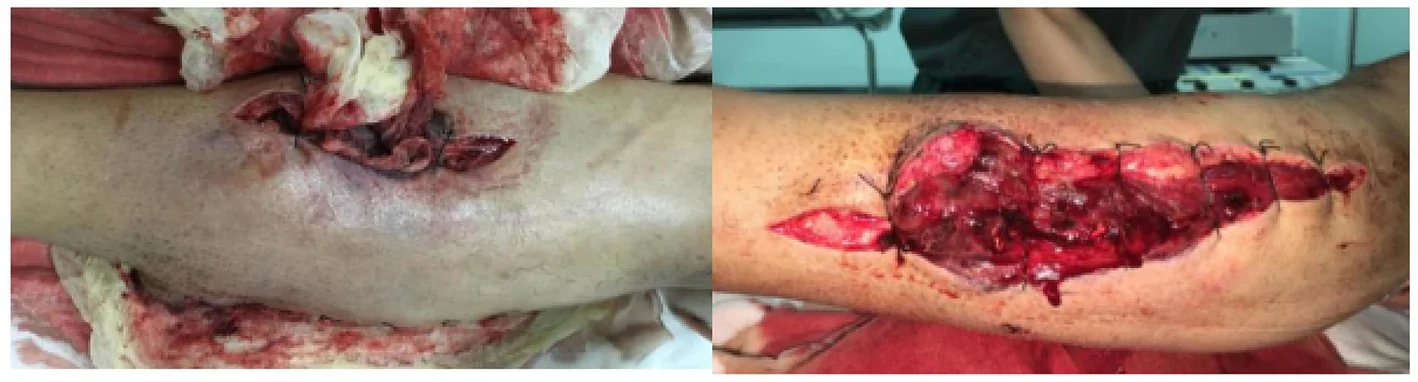
The patient underwent a simple debridement procedure at a local hospital before being transferred to our institution. The first image shows the bullet entry wound, which is relatively regular in shape with neat edges and a small entrance. The second image depicts the bullet exit wound, which is irregular in shape, with everted tissue and a larger exit.

**Figure 2:**
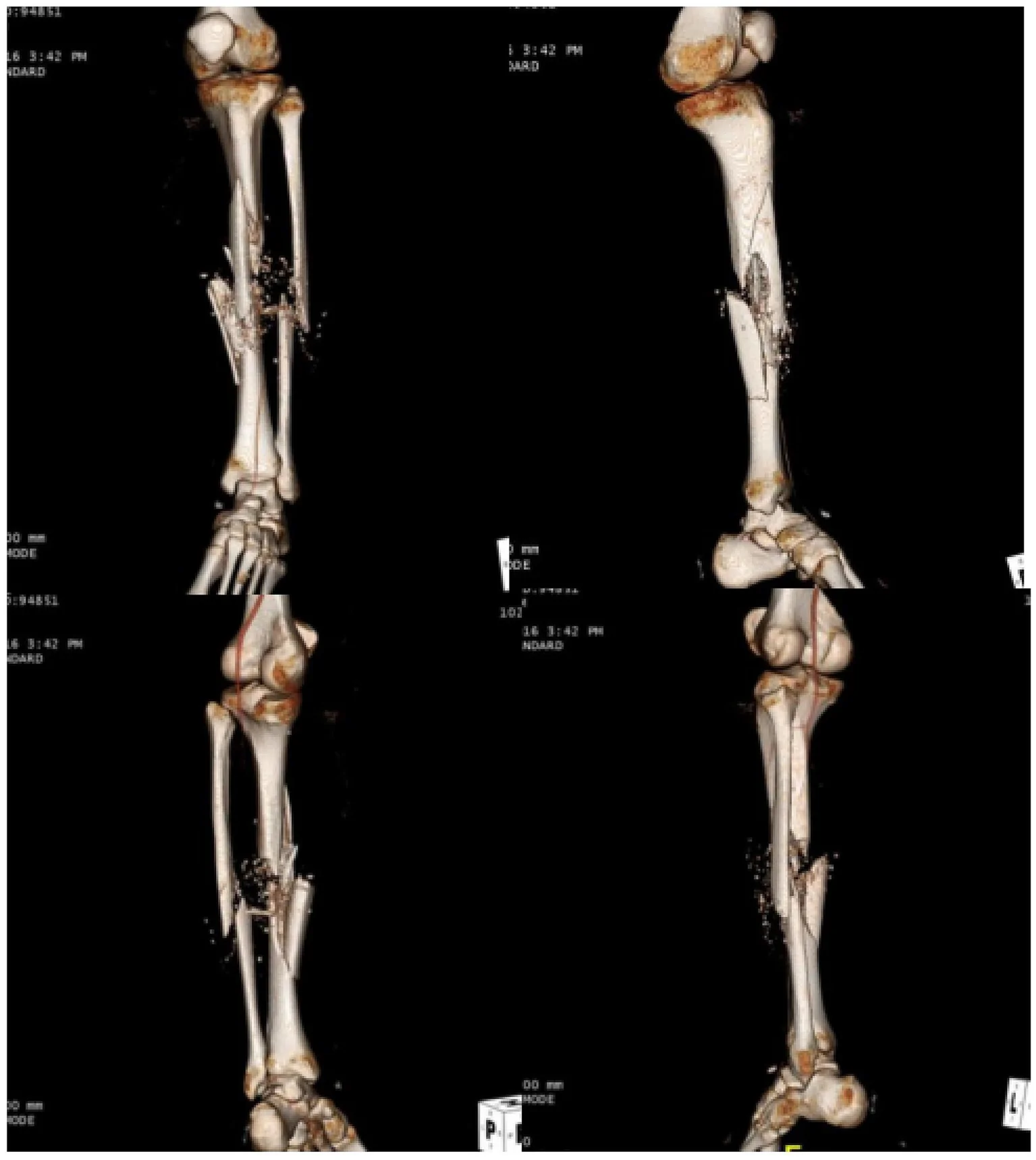
Post-injury three-dimensional CT imaging revealed a comminuted fracture of the tibia and fibula, with a significant amount of bullet fragments visible at the fracture site.

In military activities, perforating injuries account for the majority of lower limb injuries, with 63.3% of these injuries caused by explosive devices and 16.3% by high-velocity gunshot wounds (7). The high-speed impact of a bullet during a gunshot wound not only results in explosive bone fracture but also produces a “cavitation effect” in the soft tissue, causing thermal and mechanical damage to the soft tissue. The combustion gases then further strip the soft tissue along tissue and fascial boundaries, causing severe destructive injuries. More importantly, the tumbling motion of the bullet and the fragments resulting from its explosion cause secondary damage to both bones and soft tissues (12), resulting in wounds that not only exhibit complex fracture types but are also accompanied by severe soft tissue injuries (Figures 1, 2). This combined injury poses a significant challenge in treatment (9, 12).

Gunshot wounds are typically accompanied by Gustilo Type III or even more severe soft tissue injuries, and these injuries invariably involve significant contamination. Therefore, gunshot wounds cannot be sutured in the first stage; instead, they require open wound drainage and observation. Current treatment measures include the use of vacuum sealing drainage (VSD) combined with negative pressure wound therapy (NPWT) (suction: −75 mmHg) to manage contaminated wounds. Regular dressing changes are necessary to remove necrotic tissue and loose bone fragments, along with radical resection to ensure thorough local debridement (12). Additionally, the use of systemic antibiotics is crucial for preventing early infectious complications and systemic antibiotics should be administered as soon as possible after suffering from the wounds. Ideally, antimicrobial agents should be administered within 3 h after suffering from the wounds. For open wounds on the extremities, cefazolin (2 g intravenous injection, every 6 to 8 h) or clindamycin (600 mg intravenous injection, every 8 h) is recommended for 1 to 3 days. If the patient has a history of allergy to beta-lactam antibacterial agents, clindamycin can be used as an alternative. Wound irrigation should be performed as soon as possible by a professional physician, using normal saline only, without the need for other additives. For Gustilo Type I, II, and III gunshot wounds on the extremities, standard volumes of 3 L, 6 L, and 9 L, respectively, are recommended; however, for more severe injuries, larger volumes may be necessary (13).

When addressing soft tissue defects, various local flaps and VSD techniques can be utilized. Due to extensive soft tissue damage and potential damage to perforating vessels in the fascial layer during ballistic injuries, perforator-based island flaps are not common in war-related patients. However, traditional pedicled flaps, such as gastrocnemius myocutaneous flaps and sural neurocutaneous flaps, are the most common reconstruction methods in the reported literature (14).

For fractures of the femur and tibia, temporary external fixation should be utilized firstly to maintain stability at the fracture ends during soft tissue recovery and to facilitate wound observation. This helps to reduce the risk of chronic infections (compared to early open reduction and internal fixation). For open fractures of the humerus and forearm, after soft tissue stabilization, temporary external fixation or splinting should be applied initially, followed by the plate and screw fixation (4, 13).

Bone nonunion and bone defects are common complications following high-energy open injuries or debridement surgeries, severely impacting patients’ quality of life. Depending on the specific conditions of bone defects, the following surgical methods can be used:

Currently, the main methods for addressing these conditions include vascularized bone grafting, Ilizarov technique, and Masquelet technique. Vascularized bone grafting is suitable for situations requiring high survival rates and rapid healing, but it involves complex surgery and potential complications at the donor site. The Ilizarov technique is applicable to extensive bone defects, without the need for additional bone grafting, allowing early weight-bearing. However, it has a long treatment duration, poor patient comfort, and a certain risk of complications. The Masquelet technique is a two-stage treatment method where bone cement is first used to fill the bone defect, forming an osteogenic structure like periosteum, followed by bone grafting on this basis. It is suitable for various types of bone defects, especially for the treatment of long bone defects, particularly those accompanied by infection. However, it requires two surgeries and depends on the quality of the induced membrane (3, 15).

The Masquelet induced membrane technique (MIMT) is an effective method for long bone reconstruction under resource-limited conditions, particularly exhibiting unique advantages in managing bone defects caused by gunshots and explosions (1, 9). The induced membrane could stimulate bone regeneration by secreting growth factors and osteoinductive factors, making it an effective treatment for large segmental defects of long bones (16). Like bone transport techniques, MIMT has achieved success in bone defect reconstruction (12). Studies have shown that MIMT demonstrates reliable efficacy in treating major bone defects in both the upper and lower extremities (17–21). This technique achieves bone defect reconstruction through a two-stage surgical procedure. In the first stage, after debridement, antibiotic-loaded bone cement is used to fill the bone defect area. The core of this stage lies in utilizing polymethyl methacrylate (PMMA) bone cement as a foreign body to induce the formation of a membrane structure with osteogenic potential. In the second stage, the bone cement is removed, and autogenous bone or bone substitutes are implanted. Under the protection of the induced membrane, bone healing is promoted. Compared with traditional distraction osteogenesis (DO) techniques, the induced membrane in MIMT provides abundant blood supply and growth factors to the bone graft, enabling the repair of bone defects in a relatively short period of time (8). Especially when managing large bone defects, MIMT demonstrates a higher success rate and better prognosis. Furthermore, this technique, to a certain extent, can overcome issues such as donor site complications and immune reactions present in traditional bone grafting methods (22).

The present study, despite its small sample size, provides valuable clinical evidence supporting the feasibility of MIMT in treating severe gunshot-related tibiofibular bone defects. The 100% bone union rate achieved in this series is comparable to or even better than those reported in previous studies on combat-related injuries (11, 12). This may be attributed to the meticulous debridement protocol and the use of vancomycin-loaded PMMA spacers, which effectively controlled local infection and promoted a favorable biological environment for bone regeneration.

Technical details and key steps of the Masquelet technique.

**Figure 3:**
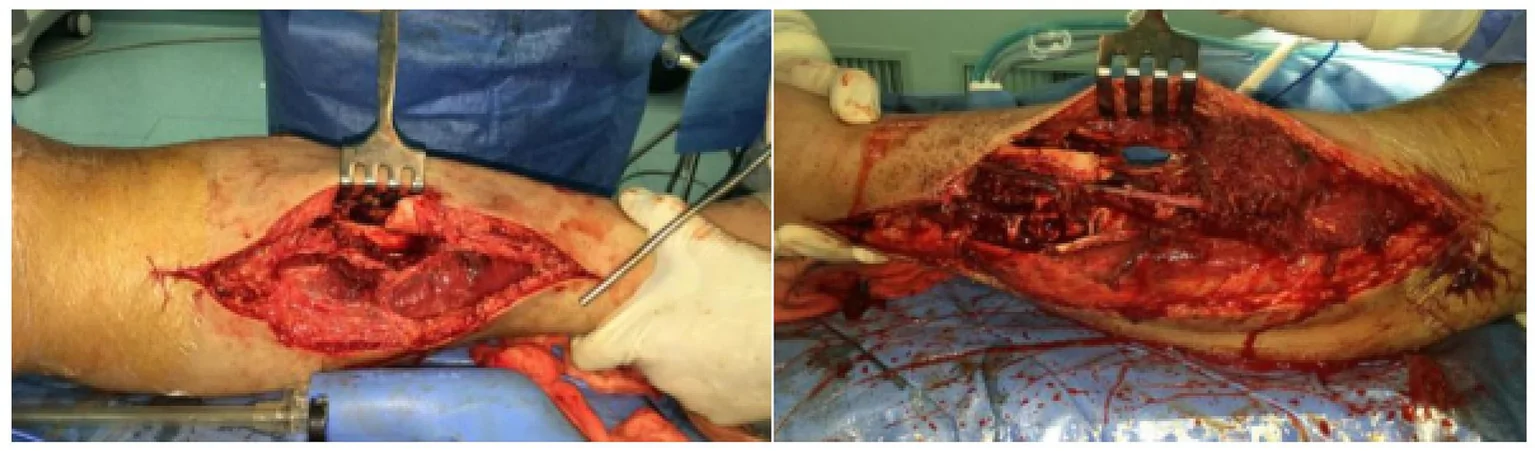
Following the initial debridement after admission, severe contamination of the soft tissues surrounding the tibia and fibula fracture site was observed. Nearly all of the muscle and fascial tissues had been removed. The anterior tibial artery was completely damaged and beyond repair, while the posterior tibial artery remained intact, allowing for continued blood supply to the distal foot.

*Stage 1*: Debridement, fixation, bone cement filling, and wound management. Firstly, thorough debridement is crucial for preventing recurrent infections. If an infection is present during the first stage, eradicating the infection (through aggressive bone and/or soft tissue debridement) is essential for successful progression to the second stage. The treatment of infectious bone defects requires thorough debridement with multiple samples sent for testing (23). All necrotic tissues, contaminants, and avascular bone tissues must be removed until fresh, bleeding bone surfaces are exposed (Figure 3) (24). Thorough debridement not only reduces the risk of postoperative infection but also creates favorable conditions for the formation of the induced membrane. Masquelet himself explained that this technique does not possess anti-infective properties, and multiple and thorough debridement surgeries are required to maintain the effectiveness of bone grafting (25).

**Figure 4:**
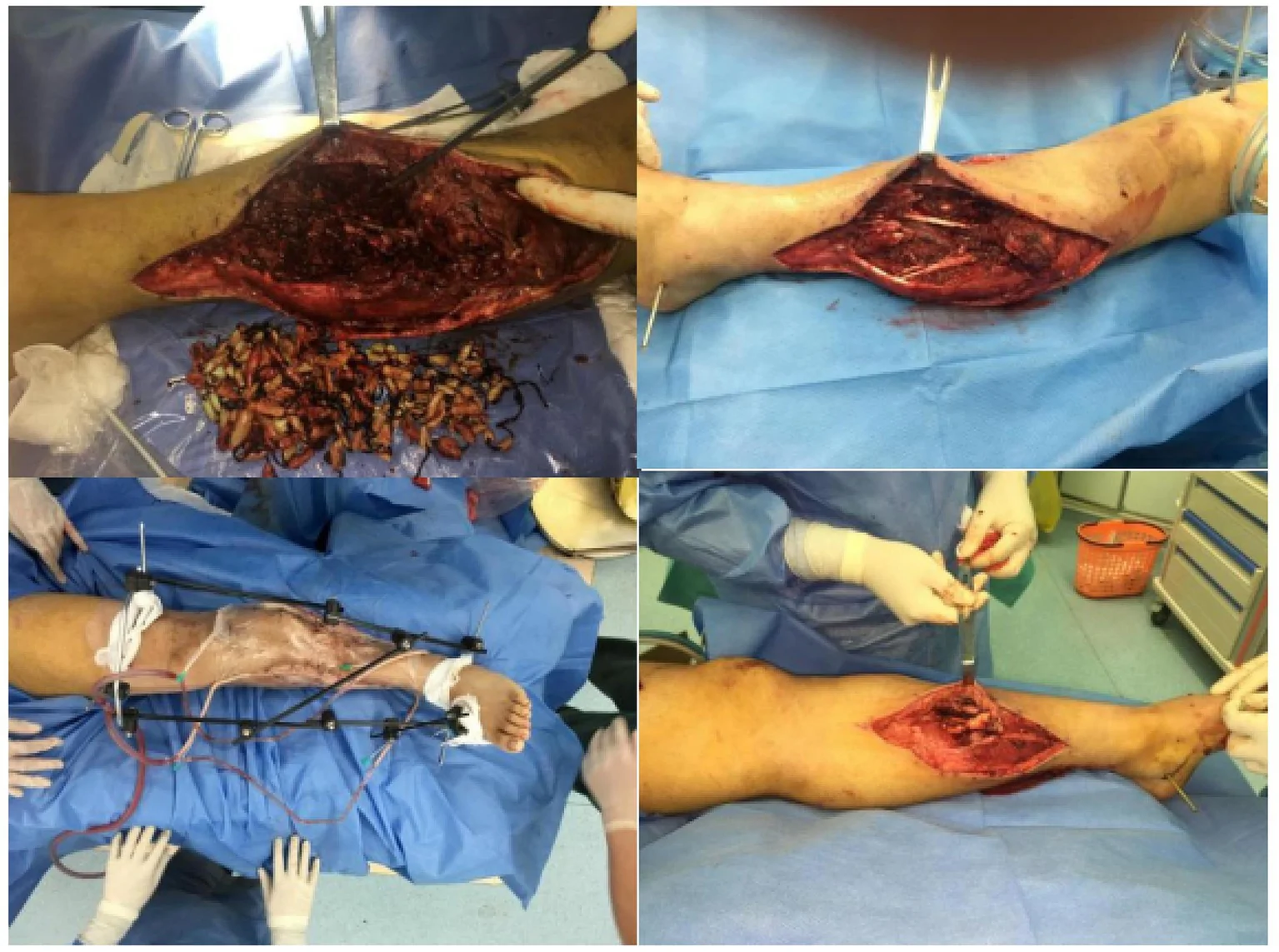
During the second debridement after admission, the vancomycin-impregnated bone cement beads were removed, and the local suspiciously contaminated and necrotic soft tissues were thoroughly debrided again. Fresh blood oozing was observed at the fracture site. Finally, the fracture was temporarily stabilized using a transarticular external fixator.

During debridement, it is important to minimize damage to the surrounding soft tissue and maintain its integrity, which is crucial for subsequent soft tissue coverage and repair of bone defects. Especially in cases of open fractures or those accompanied by severe soft tissue damage, early soft tissue reconstruction (for example, using a VSD device) can accelerate wound healing and lay the foundation for the second stage of treatment (26). During this period, bone cement beads with mixed antibiotics can be used for wound occlusion and drainage to maintain slow local antibiotic release, further protecting the wound and reducing the incidence of infection (Figure 4).

In addition to thorough debridement, ensuring adequate soft tissue coverage is also necessary. If soft tissue coverage is inadequate, additional surgical steps, such as flap transfer or skin grafting, may be required to provide coverage (27). Microsurgical techniques combined with the Masquelet induced membrane technique can effectively control infection and promote bone regeneration in the treatment of open tibial fractures. Firstly, a Masquelet-induced membrane is formed through negative pressure treatment and skin and soft tissue repair, and then flaps are designed according to the local soft tissue condition, with the use of the free anterolateral thigh flap if necessary (28, 29).

**Figure 5:**
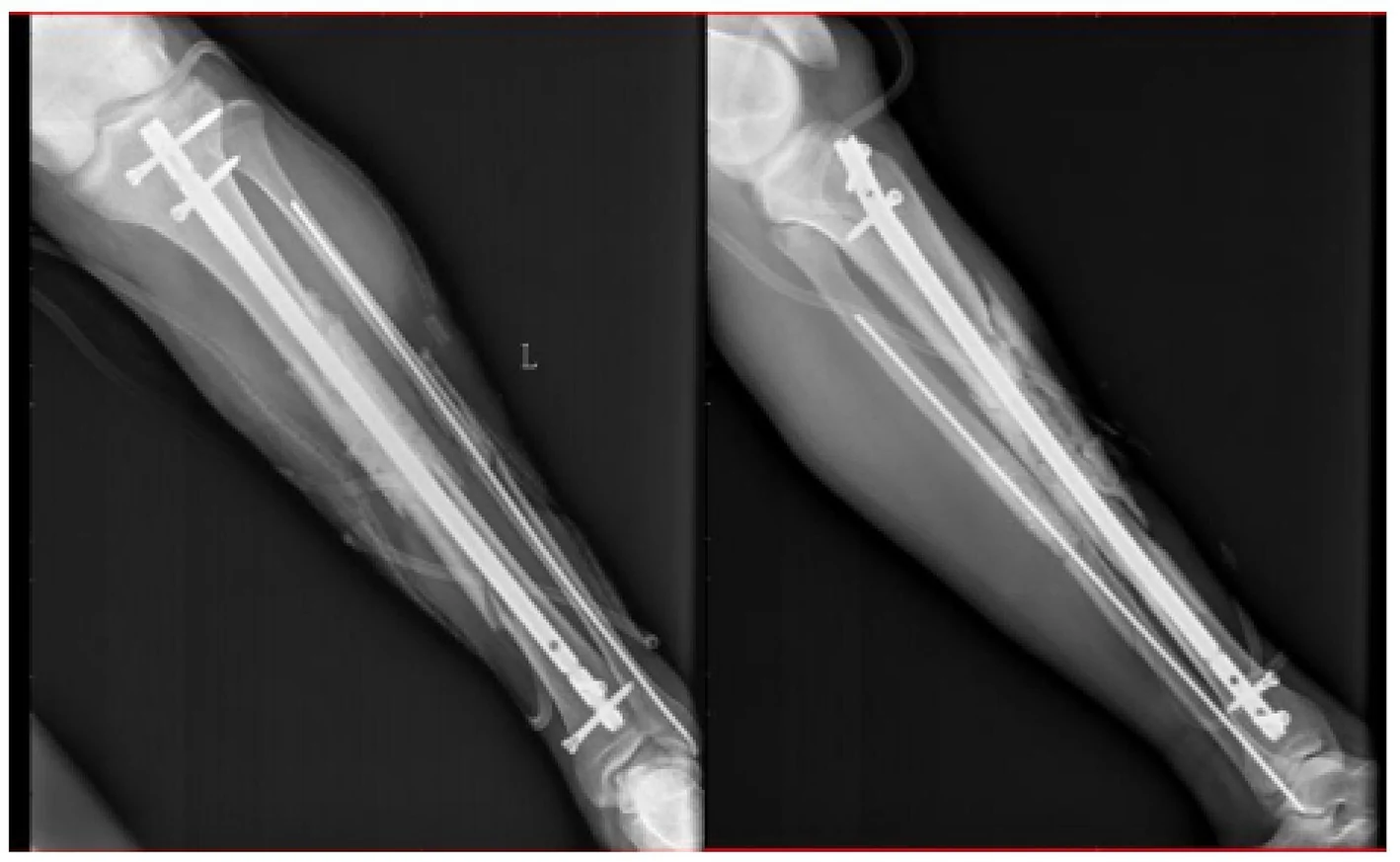
After the third debridement post-admission, the external fixator was replaced with internal fixation using antibiotic-coated intramedullary nailing technique. The bone defect area was again protected with antibiotic bone cement spacer. This image shows the immediate post-operative X-ray of the affected limb.

The selection of fixation types should consider the revascularization and later corticalization of the graft. Clinically, there are three types of fixations: external fixation, internal fixation (plates and locking screws), and intramedullary nails (IMNs). External fixation can prevent infection, is multifunctional and easy to use, but poses a higher risk of pin tract infection. Internal fixation provides high stability and minimizes the impact on defects. Studies have shown that when using the Masquelet technique for treating tibial and femoral bone defects, IMN fixation is the preferred option. Compared to plate fixation, IMN fixation can significantly reduce the reoperation rate and the need for multiple bone grafting procedures, decrease complications (30), and enable patients to resume weight-bearing faster (31). Additionally, the surgical technique involving antibiotic cement-coated intramedullary nails offers greater advantages in the treatment of severe open wounds accompanied by infection (Figure 5) (25).

PMMA is used as a bone cement spacer to maintain the space of bone defects and prepare for subsequent bone grafting. Its composition and morphology both affect the quality of the induced membrane and bone healing. Studies have found that materials such as PMMA, titanium, polypropylene, silicone, polycaprolactone (PCL), and calcium sulfate can induce the formation of membranes. PMMA spacers exhibit optimal performance in terms of the quality of the induced membrane and osteogenic potential (32). The type of cement commonly used in the Masquelet technique for bone reconstruction is PMMA cement, which serves as a spacer to induce the formation of membranes and can effectively prevent fibrosis. Moreover, PMMA cement can maintain the bone grafting space, bridge the fracture ends of bone defects, maintain the stability of the fracture site, and provide favorable conditions for subsequent bone grafting (25).

When implanting bone cement, the original shape of the bone defect should be maintained while the volume should be maximized as much as possible to provide sufficient space. It is recommended to use a 50-ml syringe for shaping, with its volume equivalent to that of the shaft at the junction of the middle and distal thirds of the adult tibia. Cylindrical spacers are commonly used clinically to adapt to bone defects (1). Spacers should be used to promote membrane formation, covering at least 1 cm of normal periosteum on both ends of the defect to keep the induced membrane away from the defect area. It needs to be shaped to achieve a good fit with the surrounding tissues before solidification. The exothermic reaction of PMMA cement is intense, so care should be taken to protect the surrounding soft tissues during the solidification process (25). It is recommended to use gloves for isolation and rinse with normal saline to remove the heat generated during the solidification process (33).

Other studies have suggested the implantation of handmade bone cement beads as an alternative to standard PMMA spacers, as they are easier to remove compared to cement blocks. Bead induced membranes may be similar to cement spacer induced membranes (34). The formation of granulation tissue can be promoted by placing antibiotic-impregnated PMMA beads followed by NPWT or open wound management (12). An innovative method is to make antibiotic nails using chest tubes, in which sterile mineral oil is added to the plastic tubing system and cold sterile saline is incorporated during cement hardening to prevent the plastic from sticking to the cement. This greatly facilitates the removal of plastic around the antibiotic nails when used to stabilize segmental bone defects (35).

The impact of whether to add antibiotics to bone cement and the influence of its surface texture on the formation of the induced membrane remains under discussion. In the early applications of the Masquelet technique, Masquelet did not recommend the use of antibiotic-containing cement in PMMA spacers (26). Masquelet et al. pointed out that antibiotic bone cement may increase bacterial resistance and adversely affect the characteristics of the induced membrane (25). However, an *in vivo* study using a rat infected femoral defect model demonstrated that antibiotics can effectively reduce surgical site infections and restore the expression of inflammatory cytokines and growth factors, such as the expression of bone morphogenetic protein-5 (BMP-5), interleukin-1b (IL-1b), IL-6, and IL-10, and tumor necrosis factor-alpha on the induced membrane. This suggests that antibiotics may play a role in bone regeneration and antibacterial capacity. The addition of antibiotics to PMMA bone cement does not increase the risk of bone nonunion, but rather shortens the healing time (36).

However, multiple studies have shown that bone cement with different antibiotics affects the formation and characteristics of the Masquelet-induced membrane. For example, PMMA spacers mixed with antibiotics such as gentamicin, clindamycin, tobramycin, and vancomycin can rapidly induce the formation of a thick fibrous membrane, which provides high concentrations of antibiotics at the defect site while reducing systemic side effects (37). Gentamicin-loaded and gentamicin-vancomycin-loaded spacers can form thicker induced membranes (IMs) (32). Gentamicin favors the formation of the induced membrane, while clindamycin may inhibit its formation (37). The use of antibiotics also requires caution, as different concentrations of antibiotics have varying effects on osteoblasts. Studies have shown that PMMA spacers supplemented with low concentrations of antibiotics can effectively control infections and induce osteoblast differentiation to promote bone formation (38). High concentrations of clindamycin can kill osteoblasts (39). Low doses of vancomycin can promote the biological activity of the induced membrane (38). The inclusion of more than 6 g of vancomycin in 40 g of bone cement can significantly reduce osteogenic and angiogenic capabilities in a rabbit radial defect model (40).

*Stage 2*: Removing the bone cement spacer, filling the bone defect with the autologous iliac bone grafts in strip form, and fixing again. The bone graft in the second stage can reactivate the biological properties of the membrane, and its main role is to prevent the absorption of the graft. The Masquelet technique is not intended for the treatment of bone infections but is used after the infection has been completely eliminated (25). There is controversy regarding the optimal timing for the second-stage surgery, which should be performed after the infection is fully controlled and the soft tissue has healed well. The widely recommended timing is 4 to 8 weeks after cement spacer implantation. However, in the present series, the actual interval was prolonged to 16 to 32 weeks. This was primarily due to the high degree of contamination characteristic of gunshot wounds, which necessitated 2 to 3 debridement procedures in Stage 1 to achieve complete infection control. Our findings suggest that in severely contaminated gunshot wounds, the infection control process may require more time, and the second-stage surgery should be performed only when all infection indicators are normalized, rather than strictly adhering to the conventional 6- to 8-week timeframe. Basic scientific research has shown that factors traditionally associated with fracture repair or bone anabolic metabolism peak within the membrane between the 4th and 8th weeks (41). Other studies suggest that the second surgery is usually performed 6–10 weeks after the first stage (25), while some recommend delaying it until the 6th to 8th weeks (42).

Despite the prolonged interval in our patients, all cases achieved successful bone union, which suggests that the induced membrane may retain its osteogenic potential beyond the 8-week window, or that the membrane can be “reactivated” upon bone grafting. This observation warrants further investigation in future studies.

In the second stage, it is crucial to handle the induced membrane with great care to maintain its integrity, avoiding damage to its vascularity, and to ensure tension-free suturing of the induced membrane following bone grafting. Debridement of the bone margin is necessary to stop bleeding, and the medullary canal should be opened to allow communication with the grafted bone. When extensive scarring obstructs the surgical approach, the use of an extended anterolateral tibiofibular approach may be considered. To correct varus deformity, a non-vascularized fibular segment can be grafted onto the medial side of the defect.

When performing intraoperative debridement, a single longitudinal incision should be made centrally through the induced periosteum, and then the cement spacer should be carefully removed to prevent damage to the bone edges and autologous periosteum. PMMA spacers are generally not able to be removed in one piece and may need to be cut into fragments with an osteotome before removal. The sclerotic bone at the end of the bone margin should be resected, and the medullary cavity should be reopened to establish communication with the graft. Larger defects may require additional filling with allograft or demineralized bone substitute in a 1:3 ratio (25). Closure of the membrane is performed to establish an enclosed space for the graft, and the remaining soft tissue is closed in layers. Imaging and clinical follow-ups are conducted until healing is achieved.

**Figure 6:**
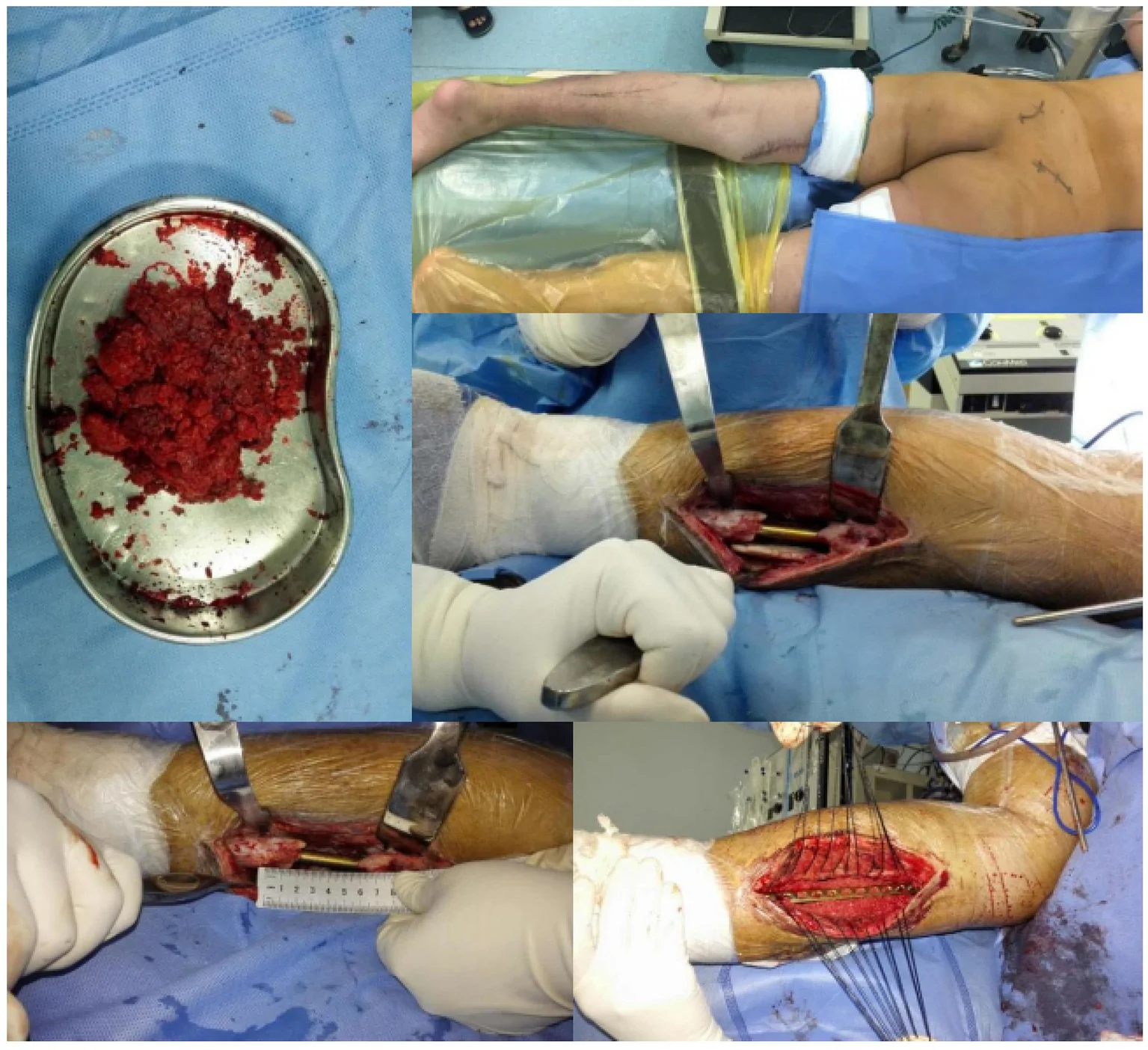
The image depicts the bone grafting procedure performed after the internal fixation was revised. Following a thorough evaluation of blood parameters (including white blood cell count, C-reactive protein (CRP), procalcitonin (PCT), erythrocyte sedimentation rate (ESR), and other pre-operative inflammatory markers, all of which were within normal ranges) and confirmation of satisfactory wound healing, an open bone grafting surgery was conducted. In this case, bone was harvested from both posterior superior iliac spines, processed into bone paste, and mixed with synthetic bone at a ratio of approximately 3:1 for grafting into the bone defect area. To enhance the stability of the intramedullary nail and minimize rotational forces at the fracture site, the surgeon reinforced the fracture with a plate. This additional fixation aimed to promote stability at the fracture site and ensure optimal healing of the grafted bone.

The gold standard for bone grafting involves the use of cancellous bone harvested from the iliac crest (Figure 6) (15), while cortical bone is not recommended as it is not conducive to revascularization by the membrane. Previous studies have shown that the volume of bone graft harvested from bilateral anterior superior iliac spines is approximately 20 cm^3^, while that from bilateral posterior superior iliac spines is about 36.9 ± 1.7 cm^3^. The use of the reamer-irrigator-aspirator system yields approximately 37 cm^3^ of bone graft (43). The cancellous bone graft must be shaped into small cubes, each measuring one millimeter in size. Due to the absence of stem cells and growth factors in allograft bone, it may be necessary to use allograft bone or demineralized bone substitutes for additional filling in a ratio of 1:3 for larger defects. The ratio of allograft bone to autologous cancellous bone in transplantation should not exceed 1/3. The small pieces of cancellous bone should be placed tightly without being crushed, ensuring the membrane cavity is filled completely, as failure to do so may result in non-union or fracture of the reconstructed segment. The membrane and attached soft tissue should be sealed on the graft, and drainage must be carried out in the membrane cavity or subcutaneous tissue; otherwise, hematoma may occur in the surgical site and lead to partial loss of the grafted bone (25). For the treatment of tibial bone defects, it is recommended that bone cement implantation be applied to fibular grafting at the same time to establish a more stable mechanical environment and increase the volume of the biological cavity. When performing bone grafting, it is advisable to include the region between the tibia and fibula as the graft site, leveraging the interosseous artery to enhance local blood supply. Additionally, by increasing the contact area between the graft and the membrane, better corticalization can be achieved, thereby accelerating bone healing. However, this method necessitates an increased amount of bone graft (6).

**Figure 7:**
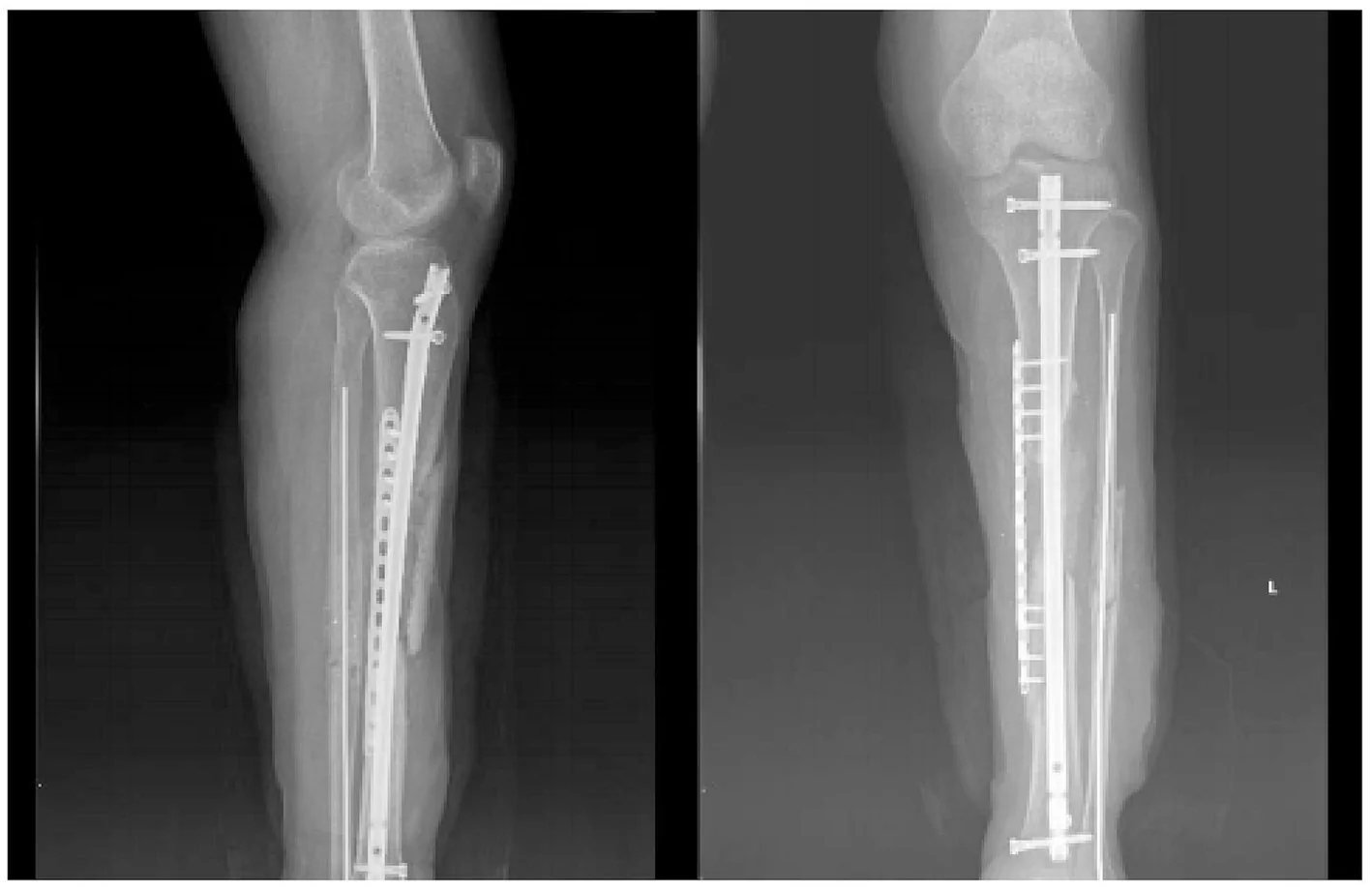
The immediate X-ray findings two days after the bone grafting procedure.

**Figure 8:**
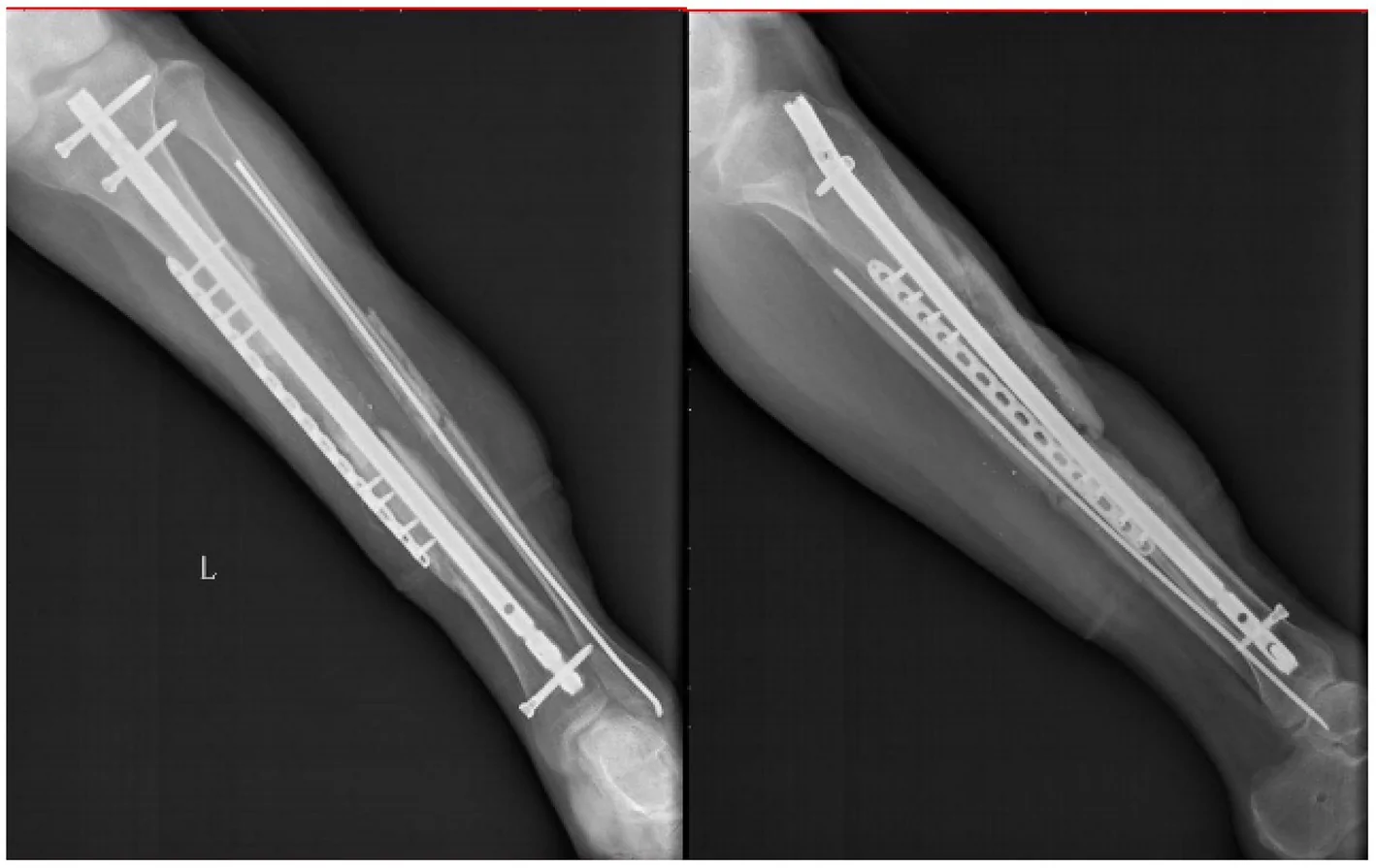
The X-ray findings one month after the bone grafting procedure.

**Figure 9:**
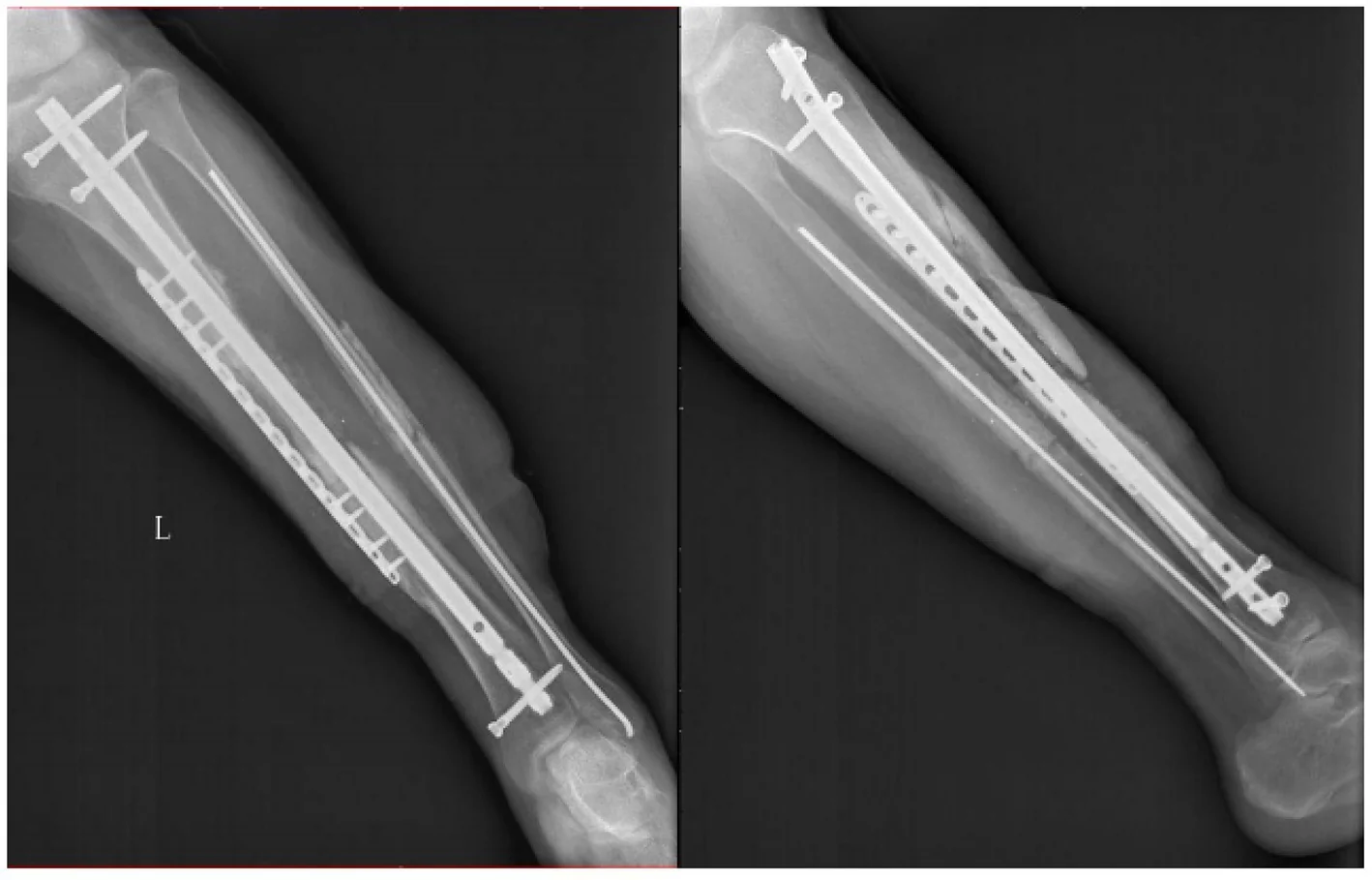
The X-ray findings two months after the bone grafting procedure, revealing partial bone resorption in the grafted area.

**Figure 10:**
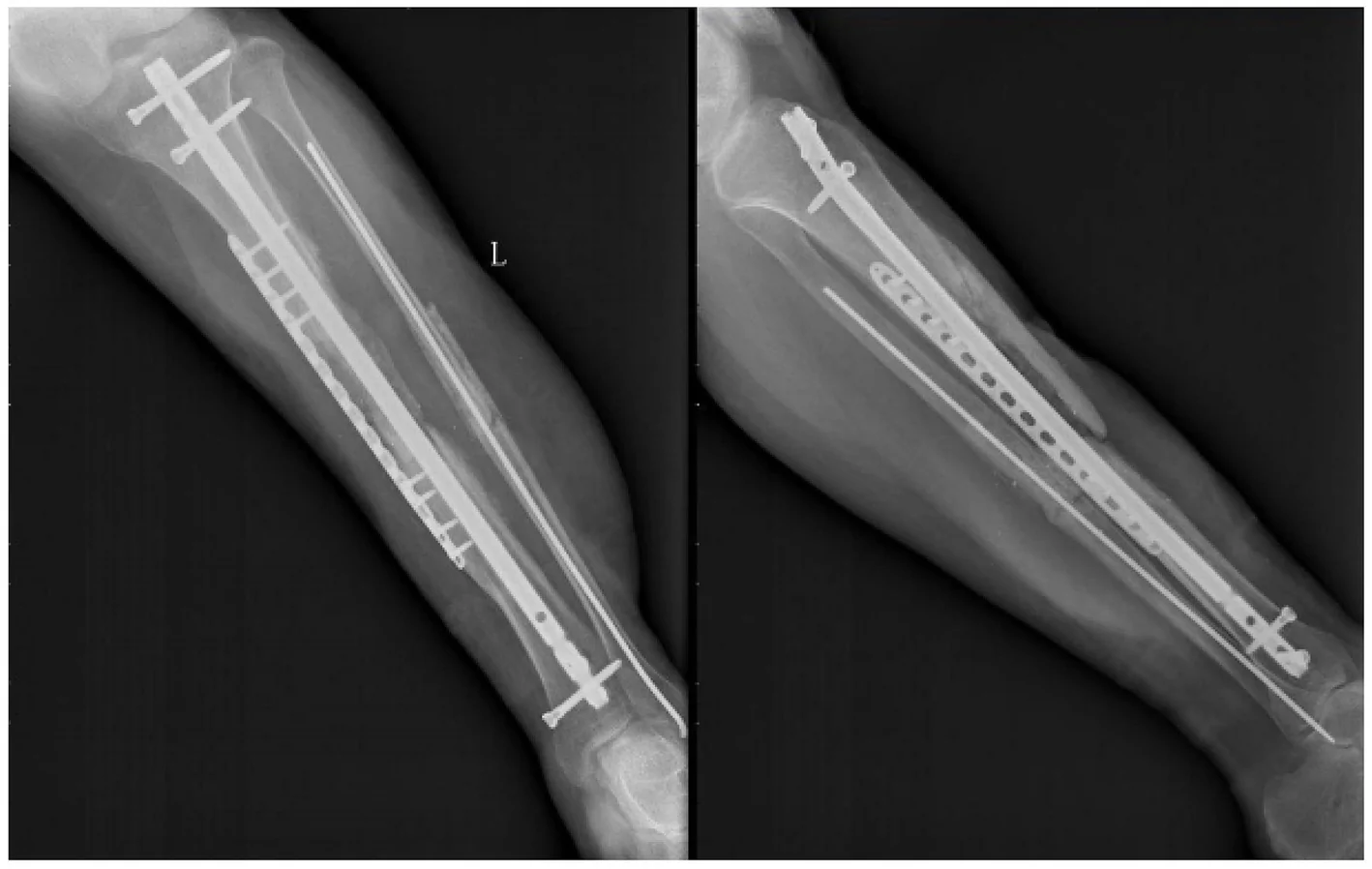
The X-ray findings three months after the bone grafting procedure, demonstrating partial bone sclerosis in the grafted area and cessation of bone resorption.

**Figure 11:**
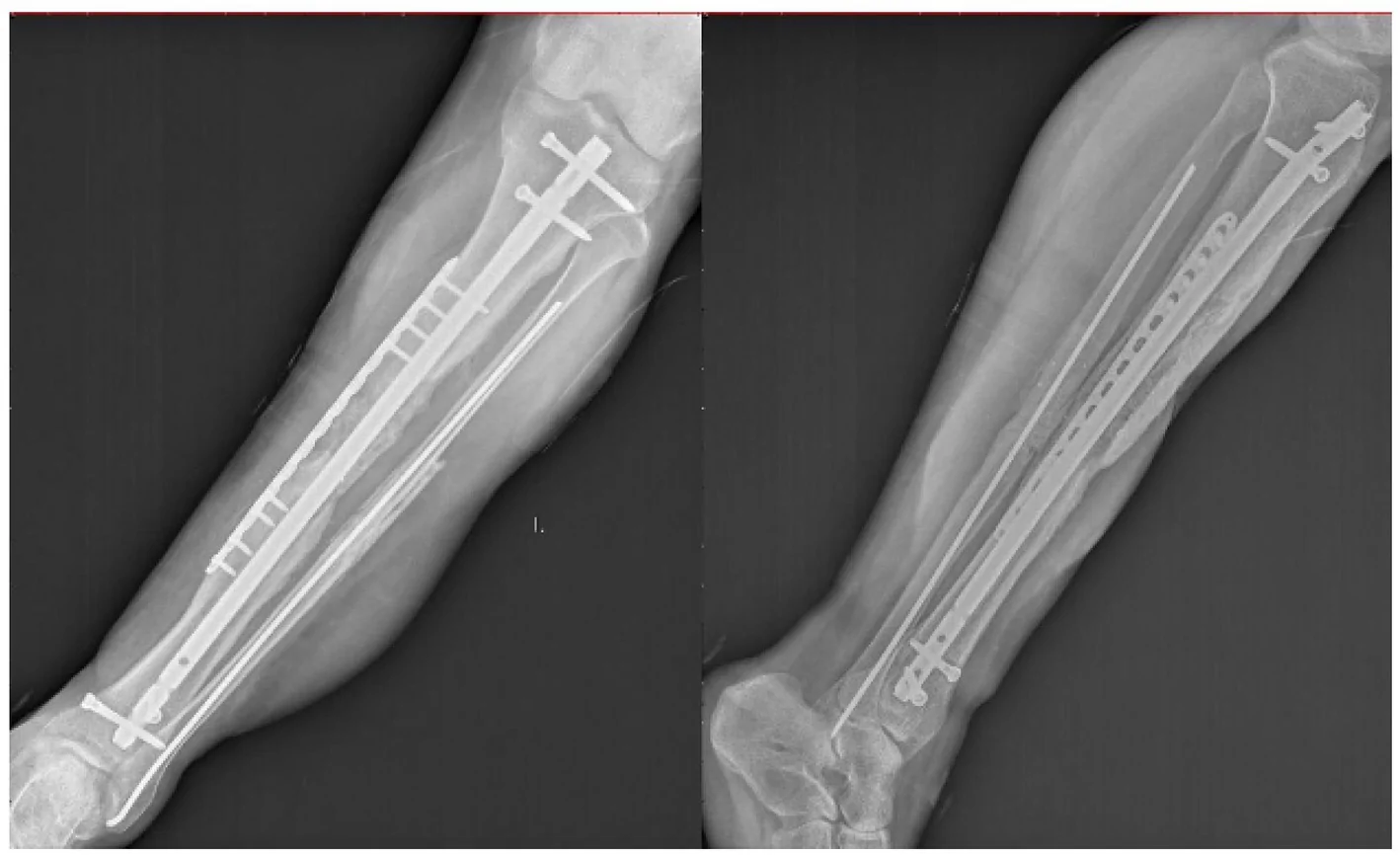
The X-ray findings 14 months after the bone grafting procedure, revealing satisfactory bone healing in the grafted area. The fracture site has largely healed, with evident cortical bone remodeling.

**Figure 12:**
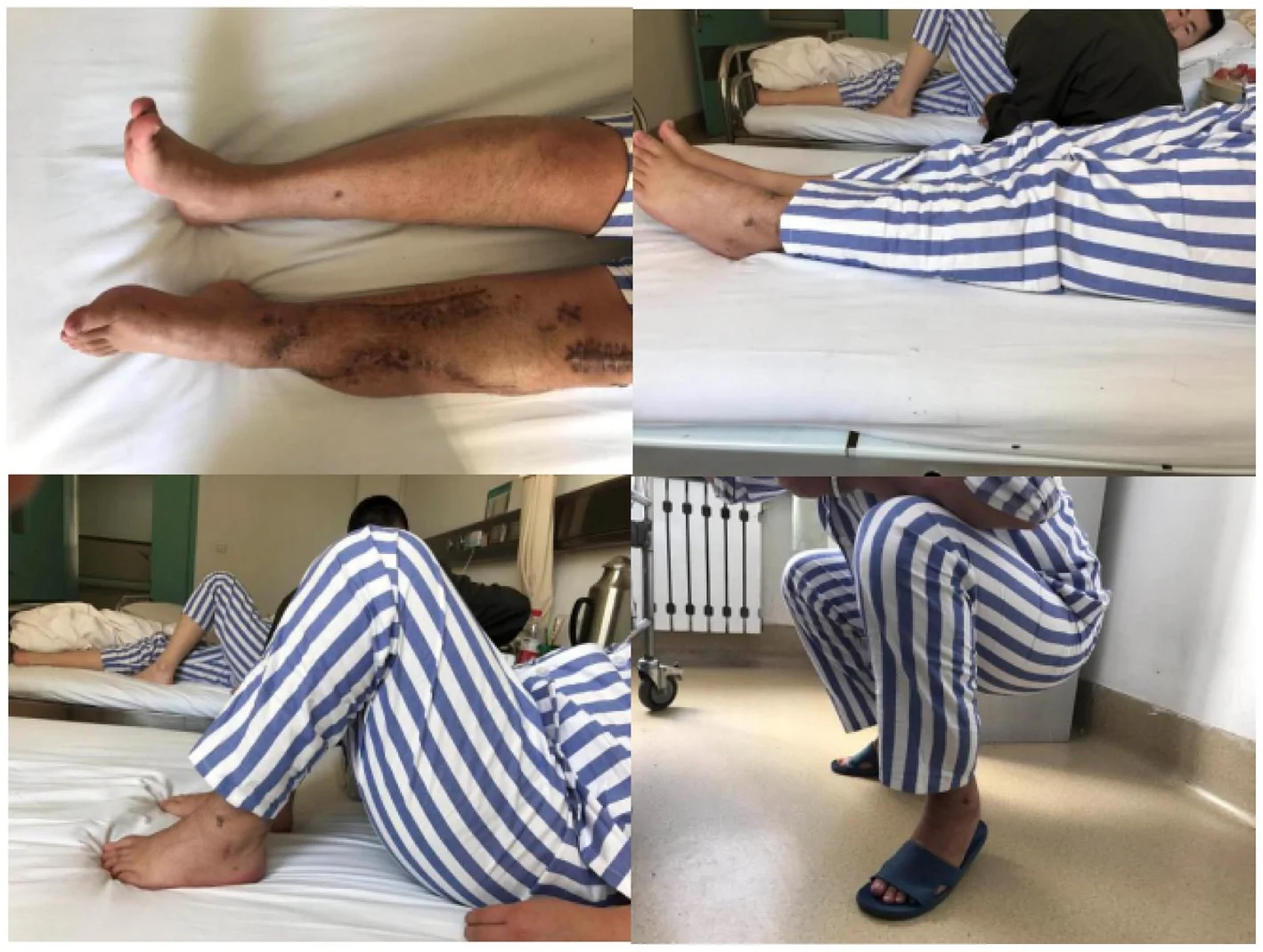
Fourteen months after the bone grafting surgery, the patient exhibited satisfactory outcomes in terms of limb appearance and range of motion in flexion and extension.

Internal fixation is preferred over external fixation in the second stage (44, 45). In a case series study on the use of the Masquelet technique for the reconstruction of segmental bone defects, despite a higher risk of septic complications, patients treated with intramedullary nail fixation exhibited advantages such as earlier weight-bearing, better biomechanical properties, and reduced bone graft volume compared to those treated with plate fixation. This is particularly true for very large tibial defects (22, 31). Studies have shown that intramedullary nail fixation can shorten healing times compared to plate fixation, allowing patients to bear weight earlier, especially in femoral fractures, potentially due to the axial load sharing and stress that facilitate cortical formation (Figures 7–12) (30).

In summary, the successful application of the Masquelet induced membrane technique relies on meticulous preoperative preparation, thorough intraoperative debridement, correct spacer design, appropriate internal fixation selection, timely second-stage surgery, and strict postoperative management. In research on the treatment of large bone defects using the one-stage Masquelet technique, significant progress has been made in electrospinning and 3D-printed polymer materials. Optimizing material properties and design may enable one-time repair of long bone defects. Although research is currently primarily conducted at the laboratory level, new polymer materials and manufacturing technologies provide a theoretical foundation and technical support for this technique (46).

Case analysis of this study. The treatment of severe open tibial fractures caused by gunshot wounds in the cases of this study included the reconstruction of bone and soft tissue defects. Compared with surgeries involving other body parts, tibial and fibular reconstruction poses specific challenges in terms of soft tissue coverage, especially infection control. This study, through a retrospective analysis, verified the significant efficacy and safety of the Masquelet induced membrane technique in the treatment of large tibial and fibular bone defects caused by severe gunshot wounds. All patients successfully completed the entire process, ranging from debridement and bone cement filling in the first stage to bone grafting in the second stage. Due to the high degree of wound contamination caused by gunshot wounds, the number of debridement increased compared to common open injuries, which significantly affected the interval between the first-stage surgery and the bone grafting surgery in the second stage.

In this study, the mean healing time was 12.5 months, which exceeded the 9-month threshold defined in our criteria for non-union. However, it should be noted that the 9-month definition applies to patients who show no signs of progressive healing at that time point. In our series, all patients demonstrated continuous radiographic evidence of callus formation and progressive bone remodeling throughout the follow-up period. Therefore, they should not be classified as non-union. The relatively prolonged healing time reflects the severity of the initial injuries and the extensive bone defects, rather than failure of the Masquelet technique.

Adequate debridement of devitalized tissues is crucial for any reconstruction method. Inadequate debridement may lead to persistent infection, osteomyelitis, and delayed amputation. Another focus of treatment is to manage limb injuries involving bone and soft tissues and to restore or preserve the continuity and function of the limb. All patients underwent standardized two-stage surgical treatment. During the first-stage surgery, all patients underwent thorough debridement, external fixation, and VSD therapy, and the bone defect area was filled with vancomycin-loaded bone cement. Satisfactory flap healing was achieved in all cases, establishing a solid basis for subsequent bone defect repair. During the second-stage surgery, the induced membranes were successfully formed in all patients, followed by the implantation of autologous iliac bone grafts in strip form in these membranes, ultimately achieving successful repair of the bone defects. During an average healing period of 12.5 months, all patients achieved bone union standards, and X-ray results showed high-quality bone regeneration and connection. This further demonstrated the effectiveness of the Masquelet technique in the treatment of tibial and fibular bone defects caused by severe gunshot wounds.

The absence of major complications in this series, despite the severity of the injuries, supports the safety profile of the Masquelet technique when performed with rigorous patient selection and meticulous surgical technique. The two cases of superficial wound infections were successfully managed conservatively, indicating that the induced membrane and local antibiotic delivery system provided effective protection against deep tissue infection.

## Conclusion

The results of this study further verify the effectiveness and reliability of MIMT in treating tibial and fibular bone defects resulting from severe gunshot wounds. This technique not only boasts advantages such as easy operation, effective infection control, and rapid bone healing, but also maximizes the restoration of patients’ joint function and quality of life. Therefore, MIMT is a therapeutic method worthy of wide promotion and application in clinical practice. Future research endeavors should aim to expand the sample size, prolong the follow-up period, and set up control groups to delve deeper into the applications of the Masquelet technique in other types of bone defects. Moreover, efforts should be made to optimize surgical timing, improve material properties and biocompatibility, promote bone tissue regeneration, and explore the possibility of one-stage surgery. However, the following limitations should be considered when interpreting the results of this study: (1) Sample size: This study only included six patients, with a limited sample size, which may affect the generality and reliability of the research findings. (2) Short follow-up duration: The average follow-up period for the patients was 13.7 ± 0.9 months, which is relatively short and may not suffice for a comprehensive evaluation of long-term efficacy and potential complications. (3) Lack of control group: This study did not include a control group, making it impossible to directly compare the advantages and disadvantages of the Masquelet technique with other treatment methods. (4) Selection bias: All patients were male and within a narrow age range, which may have introduced a certain degree of selection bias. (5) Single-center design: The study was conducted at a single institution, which may limit the generalizability of the findings. Therefore, the conclusions should be interpreted with caution, and larger-scale prospective studies with control groups are needed to validate these findings.

## Data Availability

The raw data supporting the conclusions of this article will be made available by the authors, without undue reservation.
